# Pharmacokinetic and pharmacodynamic drug interactions of carbamazepine and glibenclamide in healthy albino Wistar rats

**DOI:** 10.4103/0976-500X.77083

**Published:** 2011

**Authors:** S. Prashanth, A. Anil Kumar, B. Madhu, N. Rama, J. Vidya Sagar

**Affiliations:** *Vaagdevi College of Pharmacy, Vishwambara Educational Society, Ramnagar, Hanmakonda, Warangal - 506 001, A.P, India*

**Keywords:** Diabetic neuropathy, P-glycoprotein, antidiabetic drug, antiepileptic drug

## Abstract

**Aims:**

To find out the pharmacokinetic and pharmacodynamic drug interaction of carbamazepine, a protype drug used to treat painful diabetic neuropathy with glibenclamide in healthy albino Wistar rats following single and multiple dosage treatment.

**Materials and Methods:**

Therapeutic doses (TD) of glibenclamide and TD of carbamazepine were administered to the animals. The blood glucose levels were estimated by GOD/POD method and the plasma glibenclamide concentrations were estimated by a sensitive RP HPLC method to calculate pharmacokinetic parameters.

**Results:**

In single dose study the percentage reduction of blood glucose levels and glibenclamide concentrations of rats treated with both carbamazepine and glibenclamide were significantly increased when compared with glibenclamide alone treated rats and the mechanism behind this interaction may be due to inhibition of P-glycoprotein mediated transport of glibenclamide by carbamazepine, but in multiple dose study the percentage reduction of blood glucose levels and glibenclamide concentrations were reduced and it may be due to inhibition of P-glycoprotein mediated transport and induction of CYP2C9, the enzyme through which glibenclamide is metabolised.

**Conclusions:**

In the present study there is a pharmacokinetic and pharmacodynamic interaction between carbamazepine and glibenclamide was observed. The possible interaction involves both P-gp and CYP enzymes. To investigate this type of interactions pre-clinically are helpful to avoid drug-drug interactions in clinical situation.

## INTRODUCTION

Diabetes mellitus is the most common cause of neuropathy worldwide, and is becoming an increasing burden in countries in which the prevalence of obesity is rising.[1] Hence, with oral hypoglycemic drugs, the addition of drugs used to treat painful diabetic neuropathy is necessary in these patients. In such a situation, there may be chances for drug-drug interaction between these drugs.

Sulfonylureas are the drugs of choice in the treatment of type II diabetes. Currently glibenclamide, a second generation sulfonylurea, was preferred in therapy because of its selective inhibitory activity toward pancreatic K+ATP channels.[2] It is well established that sulfonylureas produce insulin secretion and improve tissue utilization of glucose at cellular level which was responsible for lowering of blood glucose level. The sulfonylureas and related drugs used in type II diabetes stimulate insulin by closing K+ATP channels in pancreatic β cells.

Diabetic neuropathy is often very painful, and the pain is frequently resistant to conventional treatments. Treatment with corticosteroids for a few weeks or months, carbamazepine, phenytoin, clonazepam, or paracetamol in combination with codeine phosphate can be useful along with glycemic control. Tricyclic antidepressants, such as imipramine or amitriptyline, are often effective.[3–12]

The concomitant administration of glibenclamide with carbamazepine in patients suffering from painful diabetic neuropathy may result in drug-drug interaction with enhanced/decreased glibenclamide activity, which is unwanted. The study is planned to establish the safety of the drug combination in animal models with respect to blood glucose level and find out the mechanisms responsible for the interaction if any.

## MATERIALS AND METHODS

### Drugs and chemicals

Glibenclamide and carbamazepine are the gift samples from Alka Pharmaceuticals (Hyderabad, India). Glucose kits of Span diagnostics were procured from local suppliers. The HPLC grade methanol and acetonitrile of Qualigens Fine Chemicals, Mumbai were procured from local chemical suppliers. All other chemicals used were of analytical grade.

### Animals

Experiments were performed with albino Wistar rats of either sex procured from Mahaveera Enterprises (Hyderabad, A.P., India), weighing between 180 to 210 g. The animals were housed in colony cages (four per cage) under conditions of standard lighting, temperature (22±1° c) and humidity for at least one week before the beginning of experiment, to adjust to the new environment and to overcome stress possibly incurred during transit. During this period, they had free access to food and water. The experiments were planned after the approval of Institutional Animal Ethics Committee (IEAC), Vaagdevi College of Pharmacy, Warangal, and A.P., India.

### Pharmacokinetic and pharmacodynamic studies in healthy albino Wistar rats

Albino Wistar rats of either sex were randomly distributed into four groups of six animals in each group. Before the experiment all animals were fasted for 18 h and water *ad libitum*. Water was withdrawn during the experiment. After collection of initial blood samples, drugs were administered in the following order.

**Table:** 

Group I_—	Control (0.2 ml of 0.5% carboxymethylcellulose [CMC] sodium; p.o.).
Group II—	Glibenclamide (3.6 mg/kg; p.o.).
Group III—	Pretreated with carbamazepine (90 mg/kg) followed by glibenclamide (3.6 mg/kg) after 30 minutes.
Group IV—	Pretreated with carbamazepine (90 mg/kg) for 14 days, 15th day administration of carbamazepine (90 mg/kg) followed by glibenclamide (3.6 mg/kg) after 30 minutes.

In this study, blood was collected from orbital sinuses after phenobarbital sodium (0.2%) anesthesia using heparinized capillaries into a micro centrifugation tubes contain a 0.1ml of 0.2% citric acid as an anticoagulant at 1, 2, 4, 6, and 8 h after treatment. Plasma was separated by centrifugation and stored at –20°C until further analysis. These samples are used to analyze for both blood glucose levels and glibenclamide. Blood glucose levels are estimated by GOD-POD method and glibenclamide was estimated by a sensitive RP HPLC method respectively.

### Bioanalytical method

Plasma glibenclamide concentrations were determined with a validated high-performance liquid chromatography (HPLC) method. Briefly, the HPLC system consisted of a Waters 717 Plus Autosampler (Waters Co, Milford, Massachusetts), a Waters 501 pump (Waters Co), and a 785 UV Detector (Applied Biosystems, Foster City, California) operated at 253nm. The stationary phase was a Waters Symmetry C18 column (250 × 4.6 mm, 5 μm, Waters Co). The mobile phase used was 25 mM sodium phosphate/acetonitrile (65:35, v/v, pH = 3.5) at a flow rate of 1.0 mL/min. Glibenclamide and an internal standard (glipizide) were isolated from plasma by liquid-liquid extraction with methanol. The organic phase was separated and evaporated, and the remaining residue was reconstituted with 250 μL of mobile phase before applied to the HPLC system. The method was validated and found to be linear over the concentration range of 0.1 to 10 μg/mL. Using a linear weighted least squares regression, the lower limit of quantitation (LLOQ) was 0.1μg/mL. The intra-assay and interassay coefficients of variation (%CV) for the 4 quality control standards (0.25, 2.00, 8.00, and 30.0 μg/mL) were ≤10.2% and 4.9%, respectively. The accuracy ranged between 96.7% and 99.1% for the plasma samples.

### Pharmacokinetic analysis

The pharmacokinetic parameters of glibenclamide were calculated using a sophisticated tool known as Win Nonlin (4.1) and the parameters includes half-life (T1/2), clearance (Cl_F), volume of distribution (V_F), C_max_, T_max_ and area under the curve (AUC).

### Statistical analysis

The data were expressed as mean ± standard deviation (SD). The significance was determined by applying one-way ANOVA. *P* values <0.05 were considered significant.

## RESULTS

**Figure 1:**
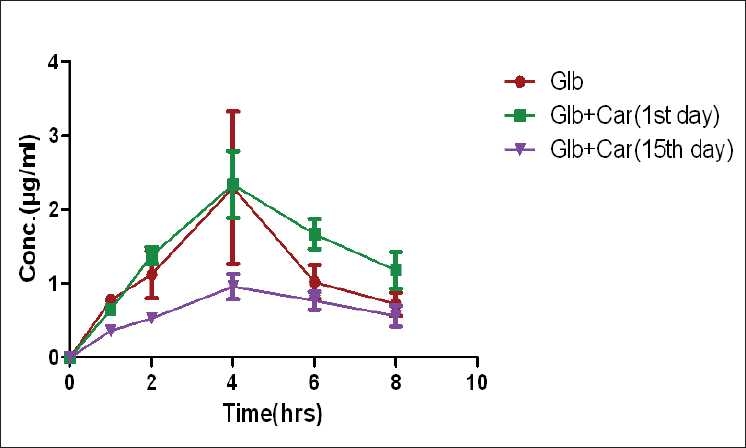
The comparison of mean ± SD plasma concentration-time profile of glibenclamide (3.6 mg/ml) following pretreatment with carbamazepine (90 mg/kg) by oral administration in healthy rats (n=6)

In the present study the plasma glibenclamide levels and pharmacokinetic parameters of glibenclamide like AUC, T1/2, CL_F, V_F, C_max_, and T_max_ were altered significantly with single- and multiple-dose treatments of carbamazepine in healthy rats and the results are shown in Table 1 and Figure 1 respectively.

**Table 1:** The pharmacokinetic profile of glibenclamide before and during carbamazepine treatment

Parameter	Glb	Glb+Car (1^st^ day)	Glb+Car (15^th^ day)
AUC (μg/ml/h)	12.96 ± 4.88	20.95 ± 3.61***	9.56 ± 2.13^NS^
K_HL(h-1)	2.24 ± 0.04	2.848 ± 0.24**	3.00 ± 0.25**
CL_F(ml/h)	42.31 ± 12.37	24.51 ± 4.51*	54.23 ± 10.85**
T_max_ (h)	3.24 ± 0.06	4.10 ± 0.34***	4.33 ± 0.36***
Cmax(μg/ml)	1.46 ± 0.51	1.86 ± 0.24^NS^	0.80 ± 0.12**
V_F(ml)	235.51 ± 45.82	147.73 ± 21.98**s	273.3 ± 41.5^NS^

* - Significant at *P*<0.05

** - Significant at *P*<0.01

*** - Significant at *P*<0.001 compared to glibenclamide control, NS-

**Figure 2:**
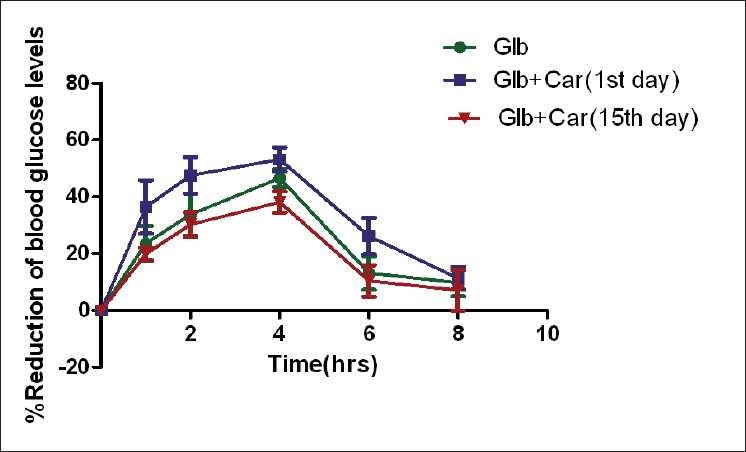
The comparison of mean ± SD percentage reduction of blood glucose-time profile of glibenclamide (3.6 mg/kg) following pretreatment with carbamazepine (90 mg/kg) by oral administration in healthy rats

The hypoglycemic activity of glibenclamide was enhanced in combination treated group than individual glibenclamide treated group (46.62±3.20% to 53.17±4.30%) at fourth hour of initial day of treatment. But in multiple dosages the combination produced less hypoglycemic activity with maximum reduction of 38.13±3.71% on 15^th^ day at fourth hour and the results are shown in Figure 2.

## DISCUSSION

Drug interactions are usually seen in clinical practice and the mechanisms of interactions are evaluated usually in animal models. We studied the influence of carbamazepine on the pharmcodynamics and pharmacokinetics of glibenclmide at therapeutic doses in healthy rats. The healthy rat model served to quickly identify the interaction. Glibenclamide is known to produce hypoglycemic activity by pancreatic (stimulating insulin secretion by blocking K+channels in the pancreatic β cells) and extra pancreatic[13] (increasing tissue uptake of glucose) mechanisms. Carbamazepine enhanced hypoglycemic effect of glibenclamide in single dosage but on multiple dosages it reduced hypoglycemic effect of glibenclamide in healthy rats.

There was a significant rise in plasma glibenclamide levels and pharmacokinetic parameters like AUC, T1/2, clearance, Vd_ss_, Vd_area_, C_max_, and T_max_ of glibenclamide with single dose treatment of carbamazepine. But on multiple dosages the plasma glibenclamide levels and pharmacokinetic parameters were significantly reduced.

In single dose studies the results showed that the co administration of carbamazepine significantly increased the AUC of glibenclamide by 38.11%. Furthermore, the T1/2, which reflects the elimination of glibenclamide, was also significantly altered by carbamazepine. These results suggest that the increased plasma concentrations of glibenclamide in carbamazepine treated rats would be caused by an increase in glibenclamide bioavailability. The possible mechanism behind this type of interaction at pharmacokinetic level may be due to inhibition of P-glycoprotein mediated transport of glibenclamide by carbamazepine in gastrointestinal tract and in renal tubules.

In multiple dosages, the results showed that the co administration of carbamazepine significantly decreased the AUC of glibenclamide by 30.86%, and the results suggest that the decreased plasma concentrations of glibenclamide in carbamazepine treated rats would be caused by a decrease in glibenclamide bioavailability. The mechanism involved in this interaction may be the induction of CYP2C9, the enzyme responsible for metabolizing glibenclamide by carbamazepine and this type of interaction is only observed with multiple doses but not with single dose. The induction process involves the synthesis of new proteins and it depends upon the half-life of inducer (carbamazepine). In the present study carbamazepine is an inducer with half-life more than 35 h and to synthesize new proteins by carbamazepine it takes more than one week in rats.

## CONCLUSION

The interaction observed appears to be pharmacokinetic interaction at absorption, metabolic and excretion levels and also at pharmacodynamic level. Whether or not similar interaction will occur in humans is needed to be investifated.
